# Editorial: Ionizing Radiation and Human Health: A Multifaceted Relationship

**DOI:** 10.3389/fpubh.2021.777164

**Published:** 2021-11-15

**Authors:** Lorenzo Manti, Dörthe Schaue, Nobuyuki Hamada

**Affiliations:** ^1^Istituto Nazionale di Fisica Nucleare (INFN), Sezione di Napoli, Naples, Italy; ^2^Radiation Biophysics Laboratory, Department of Physics “E. Pancini,” Università di Napoli Federico II, Naples, Italy; ^3^Department of Radiation Oncology, David Geffen School of Medicine, University of California at Los Angeles (UCLA), Los Angeles, CA, United States; ^4^Radiation Safety Unit, Biology and Environmental Chemistry Division, Sustainable System Research Laboratory, Central Research Institute of Electric Power Industry (CRIEPI), Komae, Tokyo, Japan

**Keywords:** ionizing radiation (IR), normal tissue adverse events, radiotherapy, BNCT (boron neutron capture therapy), FLASH, radiosensitizer, nanoparticles

Ionizing radiation is indispensable in medicine (diagnostically and therapeutically) and industry, and humans are exposed to ionizing radiation (IR) at varying doses and dose rates. This Research Topic, entitled “*Ionizing Radiation and Human Health: A Multifaceted Relationship*,” presents the opportunity to gather novel insights into what is essentially a very diverse field, encompassing several disciplines and broad expertise. It was proposed with the aim to collect studies with relevance to human health, such as cancer or non-cancer risk associated with IR exposure, along with up-to-date perspectives on strategies to increase the therapeutic benefit for the treatment of malignant or benign diseases, studies with a focus on innovative aspects in radiation protection or mitigation and radiotherapy, and animal-based or mathematical models for a better understanding of radiation responses at cellular, tissue/organ, or systemic levels. This Research Topic consists of 27 papers (three Review articles, one Mini Review article, one Perspective article, 20 Original Research articles, one Methods article, and one Brief Research Report article) published in the Radiation Oncology section of Frontiers in Oncology or in the Radiation and Health section of Frontiers in Public Health. Of these, many papers were submitted in conjunction with the 45th Annual Meeting of the European Radiation Research Society (ERRS) held in Lund, Sweden on September 13–17, 2020. Provided below is a brief outline of these papers.

There have been immense developments in various radiotherapeutic modalities. Of these, boron neutron capture therapy (BNCT) is one of the most effective therapeutic modalities for treating locally invasive and/or radioresistant malignant tumors thanks to the large neutron-capture cross sections of ^10^B along with the short ranges of their high-linear energy transfer (LET) secondary particles (1). Maliszewska-Olejniczak et al. review molecular mechanisms of DNA damage response and repair during BNCT. Bláha et al. propose an approach conceptually similar to that of BNCT, termed proton boron capture therapy (PBCT), as a strategy to make proton therapy amenable toward radiotherapy-resilient tumors, toward which proton therapy is of little avail in spite of its physical advantages in terms of tumor-conformed precision in dose deposition and sparing of normal tissue. PBCT would exploit the highly DNA-damaging secondary particles generated by the reaction between low-energy protons and ^11^B.

There is a revival in the interest of using ultra-high dose rate IR exposure, a field that has been long neglected, but has come under the public eye for the promising results shown by the so-called FLASH radiotherapy. This consists of administering therapeutic doses at very high dose rates (over 40 Gy s^−1^) which has been shown to increase sparing of normal tissues, possibly due to oxygen depletion and transient hypoxia while unchanging local tumor control, thereby improving the therapeutic index over radiotherapy carried out at conventional dose rates (i.e., at a few Gy min^−1^). Adrian et al. report that a FLASH effect appears at low doses under normoxic conditions for several cell lines *in vitro* with Konradsson et al. adding their experience of veterinary FLASH radiotherapy in canine patients. Radioimmunotherapy is an increasingly used strategy. Medler et al. define radioimmunogenic tumors that are responsive to immunotherapy combined with radiation. Lai et al. show that tumor immunogenicity is the dominant characteristic that could predict the abscopal effect of local radiotherapy. Bermúdez-Guzmán et al. address the question of circadian-based therapy and the timing of radiotherapy for treatment outcome. Paunesku et al. report on the use of X-ray fluorescence microscopy to evaluate the location of microspheres and radionuclides in liver and tumor samples following radioembolization in animal models of hepatocellular carcinoma. Li et al. performed a longitudinal quality-of-life study during concurrent chemoradiotherapy and survival in patients with advanced nasopharyngeal carcinoma.

Tumor radiosensitization is very important to improve the therapeutic index, and several novel physics and biology-based strategies are explored. To this end, Cunningham et al. demonstrate sensitization to protons by very small gold nanoparticles. Miles et al. review mouse double minute (MDM) inhibitors as radiosensitizers in the context of glioblastoma, while Nikolakopoulou et al. propose inhibitors of the Ataxia telangiectasia mutated kinase (ATM), ataxia telangiectasia-Rad3 related kinase (ATR), and the checkpoint kinase 1 (Chk1) as radiosensitizers.

In terms of modeling of IR responses at a cellular and subcellular level, Guardamagna et al. propose a unified molecular model of the chain of events initiated by radiation to interpret all experimental results in cancer cells. Rudigkeit et al. propose a deep-learning algorithm to detect and analyze cells during phase-contrast microscopy. Finally, McMahon and Prise put forward the application of a mechanistic DNA repair and survival model (Medras) for intrinsic radiosensitivity, relative biological effectiveness, and dose rates.

Normal tissue reactions following radiation exposure (e.g., of brain and heart) remain a significant concern (2–4). Zhao et al. propose global functional connectivity density to predict radiation encephalopathy at a pre-symptomatic stage. Grigorieva reviewed our current understanding of radiation effects on the brain extracellular matrix. Azimzadeh et al. show data for proteomic change in the serum following local heart irradiation in mice. Montay-Gruel et al. demonstrate that systemic delivery of extracellular vesicles derived from human embryonic stem cells ameliorates radiation-induced normal tissue complications in the lung. Yang et al. estimated the risk of second primary rectal cancer following radiation therapy for pelvic cancer. Zhao et al. report that miR-486 inhibits the proliferation of lymphoma cells and tumorigenesis induced by radiation. In addition to the aforementioned studies on tissue level changes, five papers addressed radiation-induced cytogenetic, or genotoxic changes. Bogdanova et al. show that DNA double-strand break repair foci persist in breast epithelial cells and lymphocytes after repeated exposure to diagnostic CT scans. Belmans et al. report that low dose irradiation leads to increases in DNA double-strand break repair foci in dental mesenchymal stromal cells. Habibi et al. demonstrate cytogenetic or genotoxic changes in blood lymphocytes following interventional cardiology procedures. Alsbeih et al. report the establishment of the national reference dose-response calibration curve of Saudi Arabia for dicentrics in peripheral lymphocytes to estimate radiation dose following accidental exposures. For the purpose of a rapid radiation triage, Maltar-Strmečki et al. propose the use of salty crackers as dosimeters. Pertinent to microbeam radiotherapy, Lobachevsky et al. assessed DNA damage response in non-targeted cells but receiving signals from irradiated cells.

The Editors of this Research Topic are grateful to all distinguished authors for their invaluable contributions and are indebted to the expert reviewers for their time, dedication, and constructive comments. We wish to acknowledge the Radiation Oncology section of Frontiers in Oncology and the Radiation and Health section of Frontiers in Public Health for the opportunity to guest edit this Research Topic. Many thanks also go to the ERRS2020 Organizing Committee for the tie-in with this Research Topic.

## Author Contributions

NH drafted editorial and LM and DS commented on it. All authors contributed to guest editing the Research Topic.

## Funding

LM acknowledges financial support from INFN-funded Call NEPTUNE (Nuclear process-driven Enhancement of Proton Therapy UNravEled) and MIUR PRIN PBCT (Proton Boron Capture Therapy) 2017XKWWK9. The work of DS was supported by the NIH with NCI R01CA226875 (PI DS) and NIAID U01AI148322 (PI DS).

## Conflict of Interest

The authors declare that the research was conducted in the absence of any commercial or financial relationships that could be construed as a potential conflict of interest.

## Publisher's Note

All claims expressed in this article are solely those of the authors and do not necessarily represent those of their affiliated organizations, or those of the publisher, the editors and the reviewers. Any product that may be evaluated in this article, or claim that may be made by its manufacturer, is not guaranteed or endorsed by the publisher.
